# RTA Occupancy of the Origin of Lytic Replication during Murine Gammaherpesvirus 68 Reactivation from B Cell Latency

**DOI:** 10.3390/pathogens6010009

**Published:** 2017-02-16

**Authors:** Alexis L. Santana, Darby G. Oldenburg, Varvara Kirillov, Laraib Malik, Qiwen Dong, Roman Sinayev, Kenneth B. Marcu, Douglas W. White, Laurie T. Krug

**Affiliations:** 1 The Ronald O. Perelman Department of Dermatology, New York University School of Medicine, New York, NY 10016, USA; Alexis.santana@nyumc.org; 2 Department of Molecular Genetics and Microbiology, Stony Brook University, Stony Brook, NY 11794, USA; varvara.kirillov@stonybrook.edu (V.K.); kenneth.marcu@stonybrook.edu (K.B.M.); 3 Gundersen Health System, La Crosse, WI 54601, USA; darby.oldenburg@gmail.com (D.G.O.); DWWhite@gundersenhealth.org (D.W.W.); 4 Department of Computer Science, Stony Brook University, Stony Brook, NY 11794, USA; laraib.malik@stonybrook.edu; 5 Program in Molecular and Cellular Biology, Stony Brook University, Stony Brook, NY 11794, USA; qiwen.dong@stonybrook.edu; 6 Applied Mathematics and Statistics, Stony Brook University, Stony Brook, NY 11794, USA; roman@rsinayev.com; 7 Biomedical Research Foundation Academy of Athens (BRFAA), Athens 115 27, Greece; 8 Biochemistry and Cell Biology Dept., Stony Brook University, Stony Brook, NY 11794, USA; 9 Department of Pathology, Health Sciences Center, Stony Brook University, Stony Brook, NY 11794, USA

**Keywords:** gammaherpesvirus, latency, reactivation, NF-kappaB

## Abstract

RTA, the viral Replication and Transcription Activator, is essential for rhadinovirus lytic gene expression upon de novo infection and reactivation from latency. Lipopolysaccharide (LPS)/toll-like receptor (TLR)4 engagement enhances rhadinovirus reactivation. We developed two new systems to examine the interaction of RTA with host NF-kappaB (NF-κB) signaling during murine gammaherpesvirus 68 (MHV68) infection: a latent B cell line (HE-RIT) inducible for RTA-Flag expression and virus reactivation; and a recombinant virus (MHV68-RTA-Bio) that enabled in vivo biotinylation of RTA in BirA transgenic mice. LPS acted as a second stimulus to drive virus reactivation from latency in the context of induced expression of RTA-Flag. *ORF6*, the gene encoding the single-stranded DNA binding protein, was one of many viral genes that were directly responsive to RTA induction; expression was further increased upon treatment with LPS. However, NF-κB sites in the promoter of *ORF6* did not influence RTA transactivation in response to LPS in HE-RIT cells. We found no evidence for RTA occupancy of the minimal RTA-responsive region of the *ORF6* promoter, yet RTA was found to complex with a portion of the right origin of lytic replication (oriLyt-R) that contains predicted RTA recognition elements. RTA occupancy of select regions of the MHV-68 genome was also evaluated in our novel in vivo RTA biotinylation system. Streptavidin isolation of RTA-Bio confirmed complex formation with oriLyt-R in LPS-treated primary splenocytes from BirA mice infected with MHV68 RTA-Bio. We demonstrate the utility of reactivation-inducible B cells coupled with in vivo RTA biotinylation for mechanistic investigations of the interplay of host signaling with RTA.

## 1. Introduction

Herpesviruses establish a dynamic infection in the host, characterized by productive lytic replication, a clinically quiescent stage of infection known as latency, and reactivation from latency. The human gammaherpesviruses Epstein Barr virus (EBV/human herpesvirus 4) and Kaposi’s sarcoma-associated herpesvirus (KSHV/human herpesvirus 8) establish latency in B lymphocytes and stromal cells; their latency programs are associated with neoplasms including lymphomas [1,2,3,4]. Cancer incidence increases with the loss of immune function and reactivation from latency is believed to play a critical role in disease progression [1,2,3,4]. Murine gammaherpesvirus 68 (MHV68/murid herpesvirus 4) naturally infects small rodents and shares many genetic and biological properties with the human gammaherpesviruses [5,6,7,8,9]. The MHV68 pathogen system provides a platform to examine the interplay of virus and host determinants that regulate viral gene expression and determine the latent or lytic fate of an infected cell.

The immediate early protein replication and transcription activator (RTA), encoded by ORF50, is a conserved gammaherpesvirus transcription factor that, for the Rhadinoviruses, is necessary and sufficient to initiate viral lytic replication during de novo infection and reactivation [10,11,12,13,14]. The RTAs of KSHV and MHV68 transactivate lytic genes by direct binding to RTA-responsive elements (RREs) in the viral genome or indirectly via interaction with cellular transcription factors [11,12,15,16,17,18,19,20]. In addition, KSHV RTA and MHV68 RTA exhibit reciprocity in driving reactivation from latency and transactivation of lytic promoters in the genomes of their counterparts [21,22]. While the RTA homolog of EBV (BRLF1) is sufficient to induce reactivation and lytic replication in some cell systems, BRLF1 typically functions in conjunction with the immediate early protein Zebra (ZTA) to activate viral lytic gene expression [14].

NF-κB signaling in the host cell orchestrates responses to input signals such as toll-like receptors (TLR), cytokines, and cellular stress to promote survival, proliferation, and inflammation [23]. Engagement of canonical and non-canonical NF-κB pathways by B cell surface receptors promotes B cell maturation and differentiation [24]. NF-κB signaling also determines the outcome of gammaherpesvirus infection in cell culture and in the infected host. Over-expression of the p65/RelA NF-κB subunit inhibits lytic gene promoter activation by RTA of MHV68 and KSHV and RTA/Zebra of EBV, while pharmacological inhibition of NF-κB promotes EBV and KSHV reactivation from latency [25]. Inhibition of canonical NF-κB activation by infection with a recombinant MHV68 expressing IκBαM, a dominant negative mutant form of the NF-κB inhibitor IκBα, has no impact on lytic replication, but leads to a severe defect in the establishment of latency B cells of infected mice [26]. Along similar lines, loss of upstream activators including CD40 [27,28] and BAFF receptors [29] or the downstream NF-κB1 subunit p50 in knock-out splenocytes of mixed bone marrow chimeric mice impairs MHV68 latency in vivo [26,30]. RTA of MHV68 usurps IKKβ activation to enhance its transcriptional activity and drive ubiquitin-mediated degradation of the p65/RelA NF-κB subunit to dampen inflammatory cytokine production [31,32]. These data are consistent with a role for NF-κB transcription factors in regulating gene expression to promote latency, a function that is counteracted by RTA upon de novo infection. However, the role of the lytic cycle-associated NF-κB complexes p65/p50 and the latency-associated subunits c-Rel/p50 as direct regulators of MHV68 gene expression is not known [30].

Toll-like receptors (TLRs) typically orchestrate innate and adaptive immune defenses against invading pathogens [33]. TLR engagement of the MyD88-IRAK-TRAF6 axis drives several downstream signaling events including stimulation of the IKK signalosome that leads to the nuclear translocation of activated canonical NF-κB subunits. In contrast to the role of NF-κB signaling in promoting gammaherpesvirus latency, engagement of TLRs has differential effects on MHV68 reactivation from latent B cell reservoirs and lytic replication in culture [34]. Lipopolysaccharide (which signals through TLR4) or CpG DNA (which signals through TLR9) trigger reactivation of MHV68 in cell culture; and lipopolysaccharide (LPS) administration drives MHV68 reactivation from latent splenocytes in vivo [35]. In contrast, TLR-7 and -9 engagement suppresses MHV68 reactivation from the S11 latent B cell line, but promotes reactivation in vivo [36]. Loss of the TLR signaling component MyD88 in knock-out mice impairs the establishment of viral latency [37]. Thus, TLR engagement is a host determinant of gammaherpesviruses latency, but the role of NF-κB signaling in this response is not well-characterized.

The interplay of RTA and NF-κB signaling is complex and dynamic. Herein we sought to delineate the role of NF-κB subunits in regulating MHV68 genes during lytic replication and reactivation from latency. To assess the impact of LPS/TLR4 engagement of reactivation of MHV-68, we generated latent MHV68+ B cell lines inducible for RTA expression. TLR4 activation by LPS treatment in combination with doxycycline-induced RTA expression significantly enhanced virus reactivation. However, our experiments in this system did not reveal a role for NF-κB sites upstream of *ORF6*, encoding the essential lytic single-stranded DNA binding protein [38], in response to LPS alone or in combination with the lytic transactivator RTA. Occupancy by RTA of the right oriLyt was also observed with an in vivo biotinylation system in LPS-treated primary splenocytes from infected mice, enabling validation of novel RTA recognition elements.

## 2. Results

### 2.1. Characterization of the Response of RTA Inducible A20-HE2 Latent B Cell Lines to Lipopolysaccharide

**Figure 1:**
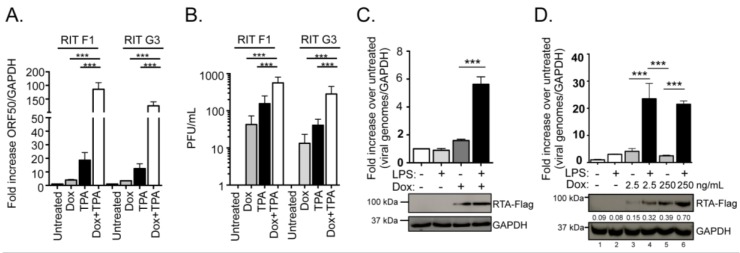
MHV68+ latent B cells reactivate upon RTA-Flag induction by doxycycline. (**A**) Quantitative PCR detection of viral DNA replication from HE-RIT cell lines 48 h after induction. The fold increase in viral genome determined by viral ORF50 normalized to GAPDH over mock treated condition shown for triplicate samples ± SD; *** *p* < 0.001, one way ANOVA, post-hoc Tukey test; (**B**) Plaque assay was performed 48 h post-treatment with Dox and TPA alone or in combination. Bars represent fold activation relative to the untreated condition for triplicate samples ± SD; *** *p* < 0.001, one-way ANOVA, post-hoc Tukey’s test; (**C**) HE-RIT G3 cell line 48 h after indicated treatment with LPS or doxycycline (Dox). The fold increase in viral genome copies (ORF50 genomic region) normalized to cellular GAPDH over mock treated condition is shown for triplicate samples ± SD; *** *p* < 0.001, one way ANOVA, post-hoc Tukey test. Immunoblot for RTA-Flag and GAPDH below; (**D**) Quantitative PCR of viral genome load from the HE RIT G3 cell line 48 h after treatment with LPS and the indicated concentration of doxycycline. The fold increase in viral genome copies (ORF46 genomic region) normalized to host GAPDH over mock treatment is shown for triplicate samples ± SD; *** *p* < 0.001, one way ANOVA, post-hoc Tukey test. Immunoblot for RTA-Flag and GAPDS below, values are the relative expression levels of RTA-FLA to GAPDH captured using a charge-coupled device camera and analyzed by ImageQuant software (v7.0; GE Healthcare).

TLR signaling via LPS enhances virus reactivation [37,39] and the IKKβ kinase promotes RTA transactivation [31]. To further characterize the contribution of host signaling to reactivation and RTA transactivation of viral genes in the context of MHV68+ B cells, we developed a cell line in which we could induce RTA expression and stimulate TLR signaling. HE2 B cell lines are tightly latent with less than 1% of cells undergoing spontaneous reactivation [40]. HE2 latent B cell lines were selected for the stable expression of an EF1α driven Tet-transactivator protein, TET3G and a tet-responsive bicistronic construct encoding C-terminal Flag-tagged RTA followed by an IRES and the tomatoRed (tdTomato) reporter gene (termed HE-RITs) (Figure S1A). Addition of doxycycline to culture media induced expression of RTA-Flag as early as 12 h post-treatment in two individual clones, HE-RIT F1 and HE-RIT G3 (Figure S1B). RTA-Flag levels remained relatively stable 48 h after doxycycline induction and TdTomato was detected by by FACs analysis (Figure S1C). Treatment of latently infected cells with 12-O-tetradecanoylphorbol-13-acetate (TPA) triggers RTA expression and reactivation [40,41]. Viral genome copies increased in both HE-RIT cell lines 48 h post-treatment with doxycycline or TPA alone, and further increased with both doxycycline and TPA (Figure 1A). The ORF65 capsid protein levels were increased when doxycycline treatment of the HE-RIT cells was coupled to treatment with the TPA (Figure S1D). Single treatment with doxycycline or TPA induced the production of infectious virus, combination treatment led to significant enhancement (Figure 1B). Single-cell analysis of GFP expression from the CMV promoter in the viral genome revealed that the percentage of cells with GFP increased two-fold with doxycycline alone, twenty-fold with TPA, and nearly forty-fold with combination treatment to 80% GFP+ (data not shown). In sum, HE-RIT cell lines are inducible for the expression of RTA-Flag. As reported for an inducible KSHV B cell reactivation system [42], RTA-expression drives a weak induction that synergizes with TPA to drive reactivation from latency.

We next investigated the impact of upstream NF-κB activation in reactivation via TLR simulation. Lipopolysaccharide is a bacterial pathogen-associated molecular pattern recognized by TLR4 that activates the canonical NF-κB signaling via the IKKβ kinase. Interestingly, doxycycline and LPS treatment at 48 h dramatically increased viral genome copies when compared to either treatment alone (Figure 1C). Immunoblot analysis detected enhanced RTA-Flag levels in HE-RIT cells treated with doxycycline and LPS compared to doxycycline alone. To investigate whether LPS enhancement of reactivation was solely the result of enhanced RTA-Flag expression, we reduced the amount of doxycycline used to treat HE-RIT cells from 250 ng/mL down to 2.5 ng/mL, either alone or in combination with LPS (Figure 1D). Treatment of HE-RIT cells with a high concentration of doxycycline achieved comparable RTA-Flag levels to that of cells treated with a very low level of doxycycline that was combined with LPS. The LPS treated condition led to a higher level of reactivation, regardless of the levels of RTA-Flag induction, (Figure 1C, lanes 4 & 5). Single-cell analysis of GFP expression in two independent experiments revealed that the percentage of cells with GFP increased from 2% in mock culture conditions to 3% with LPS alone and 5% with doxycycline alone, yet increased over five-fold to 11% GFP+ when combined (data not shown). Taken together, these data indicate that LPS treatment enhances reactivation in response to RTA-Flag induction.

**Figure 2:**
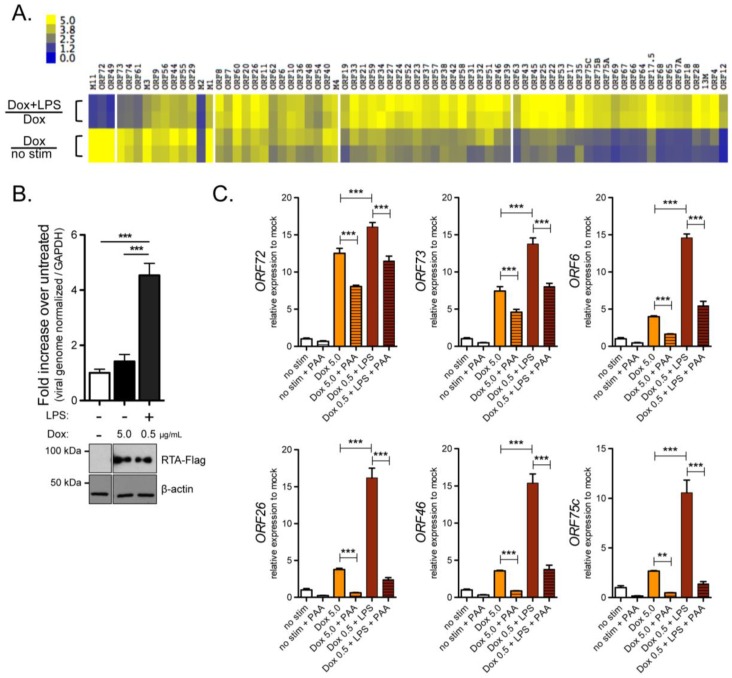
LPS treatment enhances lytic gene expression induced by RTA expression. (**A**) Global viral transcript analysis by RNAseq. RNA was isolated from HE-RIT G3 cells either untreated (no stimulation) or 24 h post-treatment with 5 µg/mL doxycyline (Dox) or a lower 0.5 µg/mL Dox in combination with 10 ng/mL LPS to ensure equivalent RNA levels. 50 bp single-end RNAseq was performed on polyA RNA by BGI Americas. Fold induction of RNA levels for duplicate samples was determined from TPM values and analyzed using the uncentered correlation for the similarity metric calculation and complete linkage for the clustering method in the Cluster program (version 3.0). The white bars indicate clustering based on optimal Silhouette scores. The heat map represents either fold change in gene expression of Dox treated samples compared to mock or Dox in combination with LPS treatment compared to Dox alone; (**B**) Quantitative PCR of viral genome load from the HE RIT G3 cell lines 24 h after treatment as described in A. The fold increase in viral genome copies (ORF46 genomic region) normalized to host GAPDH over mock treatment is shown for triplicate samples. Immunoblot for RTA-Flag and beta-actin below; (**C**) Quantitative RT-PCR analysis of the indicated genes in triplicate samples treated for 24 h with 5 µg/mL Dox or a lower 0.5 µg/mL Dox in combination with 10 ng/mL LPS, alone or in combination with 100 ug/mL PAA (Sigma). Bars represent the fold induction in transcripts within the indicated genomic region relative to GAPDH by the ∆∆C_T_ method. For B and C, bars represent fold activation relative to the untreated condition for triplicate samples ± SD; ** *p* < 0.01; *** *p* < 0.001, one-way ANOVA, Tukey’s post-test.

Given the impact on viral DNA levels, we applied RNAseq to examine for genome-wide changes in viral gene expression in response to doxycycline-induced RTA-Flag, alone or in combination with LPS. The fold change in viral gene expression when comparing doxycycline-induction to cells without stimulation and the fold change in expression of the dual LPS- and doxycycline-treated cells compared to doxycycline alone was examined by hierarchical analysis (Figure 2A). Five major clusters of genes were identified, excluding *ORF50*, which was directly responsive to doxycycline and induced to the highest levels as expected, and *ORFs 30* and *47*, which were inconsistent between independent experiments. Genes in two clusters such as *ORFs 49*, *72*, *73*, *74*, and *M11* were directly responsive to RTA induction. There was only a slight increase in their transcript levels in response to LPS and doxycycline compared to doxycycline alone. Other genes exhibited a slight response to doxycycline-induced RTA, and clustered by their degree of fold-enhancement by LPS in combination with doxyxycline. Genes in the late kinetic class of gene expression such as *ORF65* and *ORF75C* were more responsive to the combination of LPS with doxycycline-induced RTA that doxycycline alone, thus most dependent on the dual treatment. As observed in prior experiments, there was a significant increase in viral DNA only in response to LPS stimulation in combination with doxycycline-induced RTA-Flag expression (Figure 2B).

Viral genes in the late kinetic class are defined by their dependence on DNA replication [43,44,45,46]. To examine whether the LPS-enhancement of viral gene expression directly activates viral gene expression, irrespective of the regulated gene cascade, HE-RIT cultures were treated with phosphonoacetic acid (PAA) to block viral DNA replication at the time of stimulation. While PAA reduced all viral genes to some extent, transcript levels of immediate early *ORF73* and early genes *ORF72* and *ORF6* were less impacted by blocking viral DNA replication than late genes (Figure 2C). Transcript levels of late genes *ORFs 26*, *46*, and *75C* upon treatment with LPS and doxycycline in combination with PAA were reduced to levels observed with doxycycline alone (Figure 2C). Taken together, the enhancement of late lytic gene expression by LPS was dependent on viral DNA replication, suggesting that LPS-driven host signaling events act early to enhance viral gene expression.

### 2.2. ORF6 Transactivation by RTA Is Independent of NF-κB Recognition Sites

**Figure 3:**
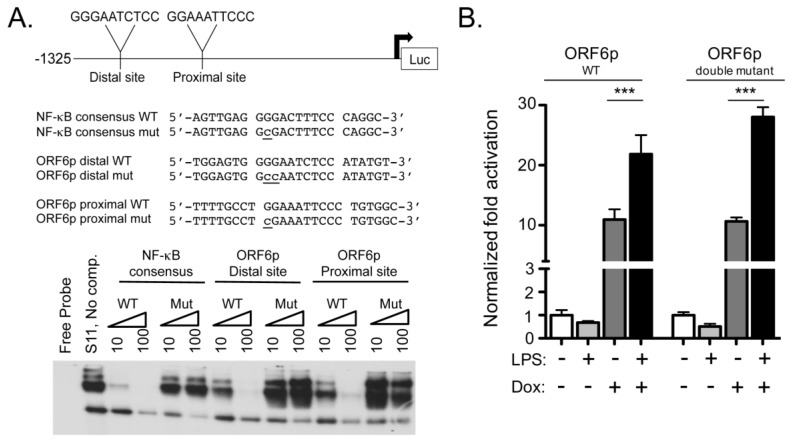
ORF6p transactivation by RTA is independent of NF-κB recognition sites. (**A**) Schematic of ORF6 regulatory region with position and sequence of distal and proximal NF-κB recognition sites. Arrow denotes ORF6 transcriptional start site. MHV68+ S11 nuclear extracts were incubated with the ^32^P-labeled NF-κB consensus WT oligonucleotide for competitive EMSA with 10- and 100-fold molar excess of unlabeled oligonucleotide with a mutant NF-κB site or ORF6p distal and proximal site oligonucleotides as indicated; (**B**) HE-RIT B cell lines were nucleofected with full-length ORF6p or ORF6p double NF-κB site mutant reporters and harvested at 24 h for luciferase activity normalized by protein content. Bars represent fold activation relative to the untreated condition for quadruplicate samples ± SD; *** *p* < 0.001, one-way ANOVA, post-hoc Tukey’s test.

ORF6 transcript levels were responsive to RTA, and were further enhanced by LPS treatment (Figure 2). We previously mapped the 5′ transcriptional start site of the ORF6 gene and demonstrated that a luciferase reporter under the control of a 1.3 kb region upstream of the start (ORF6p) is responsive to both MHV68 infection and co-transfection with the viral replication and transcription activator RTA [41]. We identified two putative NF-κB recognition sites located in the distal and proximal ORF6 regulatory region. Competitive EMSA using nuclear extracts from latent S11 B cells demonstrated competition by the WT oligonucleotides but not by oligonucleotides with mutations in either the distal or proximal NF-κB consensus sites in the ORF6 promoter (Figure 3A). Antibodies against the NF-κB subunits were used to query the composition of the complexes in nuclear extracts from lytically infected fibroblasts and the latent S11 B cells. Alterations in the mobility of the complexes upon incubation with antibodies to single or multiple subunits indicated that p65 and p50 subunits comprise the complex bound to the NF-κB binding sites in lytic extract (Figure S2A) while p50 and cRel subunits recognize the NF-κB binding sites of the ORF6 promoter in latent extracts (Figure S2B).

We sought to examine if the putative NF-κB sites in the ORF6p were responsive to LPS. First, to map the minimal RTA responsive region of the ORF6p upon de novo RTA expression, HE2 latent B cells were nucleofected with 5′ truncation mutants of the ORF6p reporter in the presence or absence of the RTA expression vector. Truncation from -909 bp to -765 bp led to enhanced transactivation by RTA. Truncation from -526 bp to -331 bp and -136 bp to -58 bp led to a progressive loss in RTA transactivation. A minimal RTA responsive element positioned 136 bp upstream of ORF6 in the HE2 latent B cell (Figure S3A) is consistent with the minimal region mapped in HEK293T cells (Figure S3B). Next, the RTA-inducible HE-RIT cells were nucleofected with the wild type ORF6p or the ORF6p double NF-κB site mutant reporter and treated with doxycycline and LPS, alone or in combination (Figure 3B). LPS treatment did not activate the WT ORF6p reporter, indicating that exposure to LPS alone is not sufficient to induce ORF6 expression. Doxycycline treatment induced the activity of each reporter, and coupling doxycycline with LPS treatment further enhanced the activity of the WT ORF6p and the ORF6p with mutations in both NF-κB sites. These data indicate that LPS treatment enhances RTA gene transactivation, but the NF-κB recognition sites in the ORF6p do not play a role in B cells undergoing reactivation.

NF-κB subunits have been found to antagonize MHV68 and KSHV RTAs in other cell systems [25,47]. To test if the NF-κB recognition sites regulate the RTA responsive region, we investigated if the proximal NF-κB site located in the -765 to -526 bp region of the ORF6p was inhibitory to RTA transactivation in HEK293T cells. No significant loss of RTA transactivation was observed upon the individual or combined mutation of the proximal or distal NF-κB recognition sites (Figure S3C). Co-transfection of NF-κB p50 and cRel or p50 and p65 subunits inhibited RTA transactivation of the ORF6p NF-κB site mutant reporters (Figure S3D). However, this inhibitory effect occurred regardless of the integrity of the NF-κB binding sites, indicating that the NF-κB subunits were not impairing RTA transactivation by occupancy of their recognition sites in the ORF6 regulatory region. Last, to determine if the ORF6p NF-κB sites influence ORF6p activation in the context of de novo lytic infection, NIH3T12 cells were transfected with the ORF6p reporter and then infected at a multiplicity of infection (MOI) 5 with the control MHV68 or MHV68-IκBαM, a recombinant virus that expresses a mutant form of IκBα which functions as a super-repressor of canonical NF-κB activation [26]. The ORF6p reporter was induced to comparable levels upon infection with either WT MHV68 or MHV68-IκBαM (Figure S3E). This indicates that ORF6p reporter transactivation by RTA is not influenced by NF-κB subunit binding during lytic infection, consistent with our findings for the RTA-inducible HE-RIT reactivation system.

### 2.3. RTA Occupancy at the Right Origin of Lytic Replication upon Induced Reactivation from Latency in Cultured B Cells

**Figure 4:**
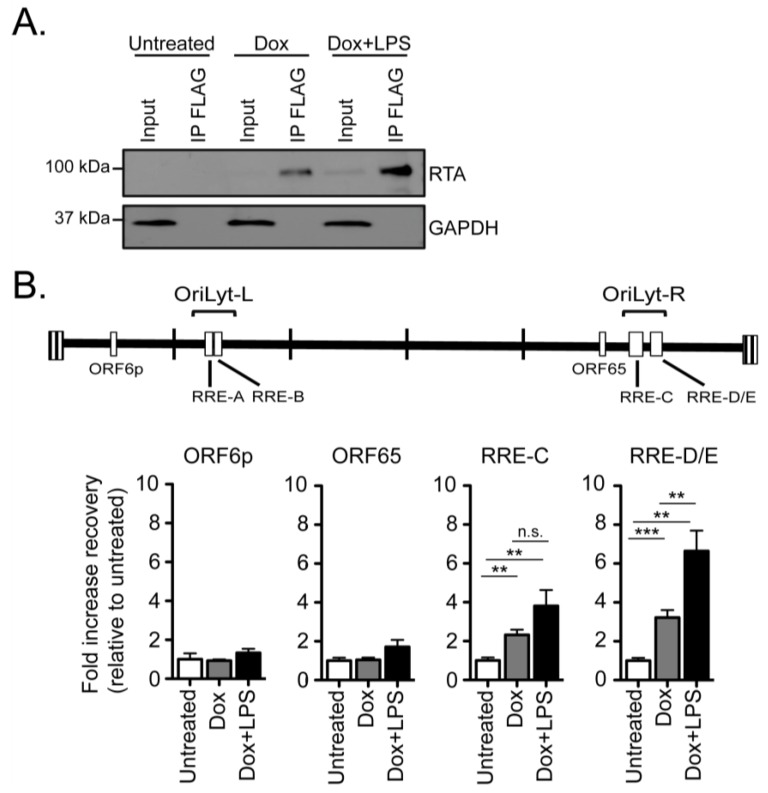
Chromatin immunoprecipitation of RTA on the right origin of lytic replication (OriLyt-R). (**A**) Western blot of immunoprecipitates recovered by anti-Flag conjugated beads in HE-RIT G3 cell lines. Input shown is 1% of total; (**B**) Quantitative PCR was used to measure the indicated genomic regions in complex with RTA-Flag; RRE-RTA recognition element [16]. Data shown as fold increase in % input recovery compared to untreated cells, triplicate samples ± SD; ** *p* < 0.01; *** *p* < 0.001, one-way ANOVA, post-hoc Tukey’s test.

To examine whether LPS enhancement of ORF6p transactivation by RTA is accompanied by an increase in the occupancy of the viral genome by RTA, we performed chromatin immunoprecipitation (ChIP) of RTA-Flag in HE-RIT G3 cells treated with doxycycline alone or in combination with LPS at 24 h post-treatment. Slightly higher levels of RTA-Flag were immunoprecipitated in HE-RIT cells treated with doxycycline and LPS (Figure 4A). Regardless of treatment condition, RTA-Flag did not pull down the minimal ORF6 0–150 bp region or the previously characterized negative control ORF65 genomic region [16] (Figure 4B). These data suggest that RTA transactivation of the ORF6p minimal promoter element may occur via an indirect mechanism that does not require direct RTA binding to ORF6p.

We next examined if RTA occupancy of the right oriLyt correlated with the increase in viral DNA replication upon LPS stimulation. Hong et al. [16] previously identified an RTA recognition element (RRE) in the left origin of lytic replication (oriLyt) that controls ORF18 expression. Based on sequence conservation, three additional RRE elements were predicted in the right oriLyt [16]. Indeed, we identified RTA-Flag complex formation with the right oriLyt upon doxycycline induction. One region spanned the predicted RRE-C and a second region 552 bp away encompassed the RRE-D and RRE-E sites that are separated by 63 bp (Figure 4B). There was a significant increase in the pull-down of RTA in complex with the right oriLyt region that spans the RRE-D/E sites upon combination treatment with LPS and doxycycline (Figure 4B), perhaps attributed, in part, to the higher level of RTA-Flag in the immunoprecipitates treated with LPS (Figure 4A). In summary, RTA interacts with both the left and right oriLyts of MHV68, and these regions encompass RREs [16]. The HE-RIT system provides the tools to characterize the interaction of RTA with the viral genome in the context of multiple reactivation stimuli.

### 2.4. RTA Complexes with the Right Origin of Lytic Replication in Infected Primary Splenocytes upon Explant Reactivation

**Figure 5:**
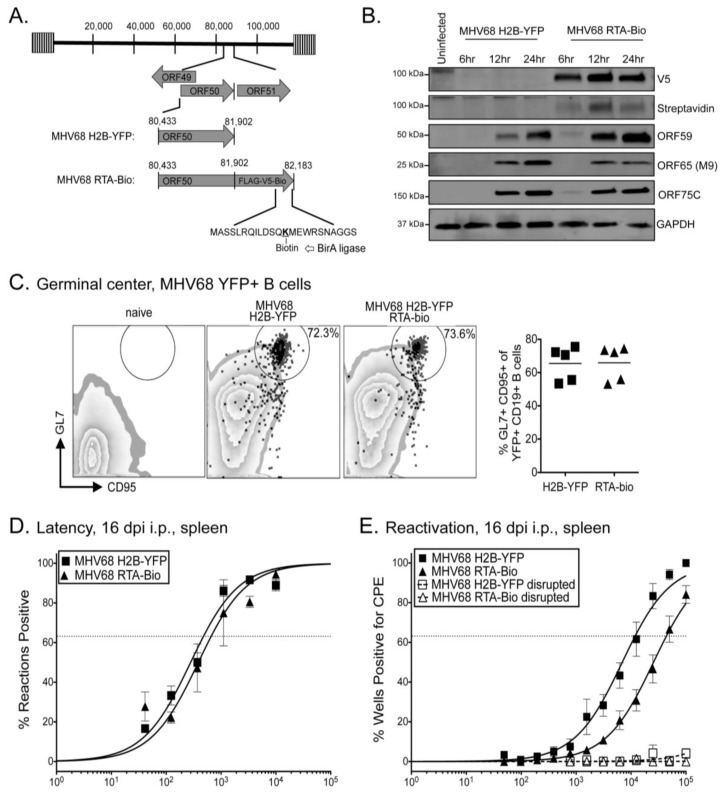
Construction and characterization of the recombinant MHV68 RTA-Bio virus. (**A**) Schematic of MHV68 RTA-Bio indicating the Bio tag insertion at the 3′ end of RTA. Biotinylation occurs on the lysine residue in the presence of BirA ligase; (**B**) Time course immunoblot analysis of RTA-Flag-V5-Bio and other viral gene products upon infection of BirA expressing MEFs with H2B-YFP or RTA-Bio viruses (MOI 5.0); (**C**) Percentage of GL7+ and CD95+ germinal center B cells in the MHV68 YFP+ population of mice infected with the indicated viruses. Left panels, black dots indicate YFP+ cells and the gate indicates the percentage of YFP+ cells that are GL7+CD95+ B cells. The individual cells are overlaid on the flow cytometry plot of total CD19+ B cells. Representative flow cytometry plot of B cell response in naïve mice provided. Right panel, summary plot of the percentage of MHV68 YFP+ cells that bear the germinal center markers GL7 and CD95; (**D**) Latency was measured at 16 dpi in the spleen, as indicated by the frequency of intact splenocytes harboring the viral genome using a limiting-dilution nested-PCR assay; (**E**) Reactivation from latency was measured by using a limiting-dilution explant reactivation co-culture assay. Dotted lines represent disrupted cells used to measure preformed infectious virus. The intersection of the nonlinear regression curves with dashed line at 63.2% is used to determine the frequency of cells that were positive for either the viral genome or reactivating virus. Graphs represent SEM of three-five independent experiments using three-four mice each.

LPS treatment of mice or primary splenocytes infected with MHV68 enhances virus reactivation from latency [35,39]. We hypothesized that LPS treatment would enhance our detection of RTA interactions with the viral genome in primary splenocytes. To test this in primary splenocytes, we generated a recombinant MHV68 virus that encodes a tagged RTA to enhance the isolation of RTA-DNA complexes from the rare population of infected cells in vivo. The RTA-Bio construct encodes MHV68 RTA with a C-terminal fusion of FLAG, V5, and a biotin acceptor sequence (Figure 5A). The biotin acceptor sequence is a 23 amino acid peptide tag that is biotinylated in the presence of the bacterial E. coli BirA protein ligase [48]. In mammalian cells, very few naturally occurring proteins are biotinylated, thus proteins covalently linked to biotin enables streptavidin-mediated enrichment from in vivo tissues [49,50]. En passant mutagenesis was performed to generate two independent recombinant viruses encoding RTA-Bio on the MHV68 H2B-YFP reporter virus backbone (MHV68 H2B-YFP-RTA-Bio.1 and –RTA-Bio.2). Single MfeI and BamHI digestion of BAC DNA and PCR amplimers confirmed genomic integrity and tag insertion, respectively (Figure S4A). In addition, whole-genome sequencing of the parental MHV68 H2B-YFP and MHV68 RTA-Bio.1 BAC DNA confirmed the sequence of the C-terminal Bio tag and did not reveal any additional mutations in the unique sequence of the genome (data not shown).

Mouse embryonic fibroblasts derived from transgenic mice that constitutively express BirA (ROSA BirA MEFs) were infected with MHV68 H2B-YFP or the MHV68 RTA-Bio.1 virus at an MOI of 5. Immunoblot analysis detected both RTA expression and biotinylation (Figure 5B). In addition, ORF59 early gene expression increased throughout the 24 h time-course for both viruses. Late gene products, the capsid protein ORF65 (M9) and tegument protein ORF75C, were detected at 12 and 24 h post-infection at comparable levels. Single-step growth curves were performed for MHV68 H2B-YFP and MHV68 RTA-Bio.1 viruses (MOI 5) in WT C57/BL6 MEFs and ROSA BirA MEFs. RTA-Bio.1 virus replication was decreased when compared to H2BYFP (Figure S4B).

Given the slight attenuation in replication observed in cell culture, we infected ROSA BirA mice with 1000 PFU of MHV68 H2B-YFP or MHV68 RTA-Bio by the intraperitoneal inoculation, a route that is less restrictive than the lower respiratory route. The peak of MHV68 latency in the spleen (~16 dpi) coincides with germinal center formation as the host responds to infection. The B cell response to infection was analyzed by flow cytometry. CD19+ B cells participated in germinal center reactions and underwent immunoglobulin class-switching to IgG2b at comparable levels in ROSA BirA mice infected with either MHV68 H2B-YFP or MHV68 RTA-Bio (Figure S5A). We next characterized the splenic B cells harboring the YFP+ B cells infected with either virus. The frequency of YFP+ CD19+ B cells was nearly equivalent between ROSA BirA mice infected with MHV68 H2B-YFP or MHV68 RTA-Bio (Figure S5B). In addition, both viruses colonized CD95+GL7+ germinal center (Figure 5C) and IgG2b+ IgD- class-switched (Figure S5C) CD19+ B cells at similar frequencies.

The ability of the virus to colonize the germinal center and class-switched B cell population following intraperitoneal inoculation suggested that latency establishment was not impacted by the C-terminal RTA Bio tag. In agreement with the flow cytometry analysis, we did not observe a change in the establishment of splenic latency based on overlapping frequencies of genome-positive splenocytes from ROSA BirA mice infected with MHV68 H2B-YFP or MHV68 RTA-Bio (Figure 5D). However, there was a decrease in the reactivation frequency of MHV68 RTA-Bio.1 from latency upon explant of splenocytes (Figure 5E). After intraperitoneal infection, latency establishment was reduced in the peritoneal exudate cell reservoir (PECs) in ROSA BirA mice infected with MHV68 RTA-Bio as compared to mice infected with MHV68 H2B-YFP (Figure S5D), and reactivation from PECs was also decreased (Figure S5E), as found for the splenocytes. Taken together, the tagged RTA does not impact splenic latency upon direct infection of the peritoneal cavity, but does diminish the efficiency of reactivation from latency, consistent with observations of decreased replication of the tagged RTA-Bio virus in cell culture. Importantly, the ability of the virus to gain access to the germinal center B cell population enabled us to next examine how LPS impacted RTA occupancy of the viral genome upon explant reactivation of a natural latency reservoir.

**Figure 6:**
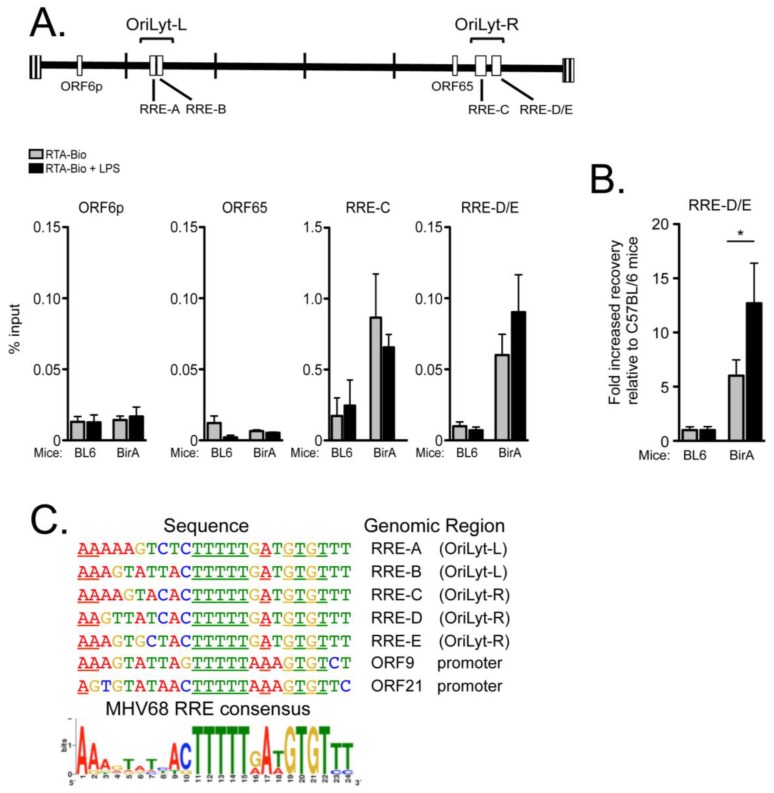
Chromatin immunoprecipitation of the right oriLyt (OriLyt-R) with RTA-Bio from primary splenocytes isolated from infected Rosa BirA mice. (**A**) C57/BL6 or BirA expressing transgenic (Rosa BirA) mice were infected at 1000 pfu and primary splenocytes harvested 16 dpi. Cells were cultured in media for 18 h with or without LPS. Immunoprecipitates using streptavidin conjugated beads were analyzed by qPCR for the indicated genomic regions and calculated as a percentage of input DNA. Graphs represent SEM of three independent experiments using two mice each; (**B**) Enrichment of RTA occupancy on the oriLyt-R upon LPS stimulation. Data represents analysis of (**A**) as the fold-enrichment in recovery from the Rosa BirA splenocytes compared to the recovery from the C57/BL6 splenocytes; * *p* < 0.05, one-way ANOVA, post-hoc Tukey’s test; (**C**) Find Individual Motif Occurrences (FIMO) was used to search the MHV68 genome for RTA recognition elements based on the RRE in the oriLyts [16]; the ORF9p site has been validated elsewhere [51]. An MHV68 consensus for RTA recognition elements is presented as a position weight matrix determined from MEME analysis.

ChIP-qPCR was performed using primary splenocytes isolated from C57BL/6 and ROSA BirA mice infected 16 days prior with MHV68 RTA-Bio via the intraperitoneal route. Primary splenocytes were left untreated or treated with LPS for 18 h after explant. ChIP using streptavidin-conjugated beads to capture biotinylated RTA was followed by quantitative PCR with two primer sets targeting the regions of the right oriLyt that comprises three closely spaced RRE sites occupied by RTA-Flag in the HE-RIT experiments (Figure 4). Splenocytes from ROSA BirA mice infected with MHV68 RTA-Bio virus were enriched for RTA occupancy on the right oriLyt, but not the ORF6 promoter or ORF65 genomic region (Figure 6A). LPS treatment led to a statistically significant increase in RTA occupancy of the right oriLyt region in the splenocytes of ROSA BirA mice in comparison to the C57BL/6 mice (Figure 6B). Thus, RTA occupancy of the right oriLyt was validated in two distinct systems, a latent B cell line inducible for RTA expression (Figure 4B) and primary splenocytes from mice infected with a virus that expresses an RTA that is biotinylated in vivo (Figure 6B).

We generated a position weight matrix based on the RREs of both oriLyts. Searching the MHV68 genome with this matrix identified 7 potential binding sites with statistically significant *p*-values and *q*-values (*p* < 1 × 10^−6^, *q* < 0.1) based on an analysis of background DNA Markov probabilities. Five of these sequences were the previously identified RRE sites in the left oriLyt [16] and the RREs in the region of the right oriLyt bound by RTA in this study (Figure 4B and Figure 6B). Two additional genomic regions were identified in regions upstream of ORF9 and ORF21 (Figure 5C). The ORF9 RRE is directly bound by RTA and mediates RTA transactivation [51], but the ORF21 RRE has not been investigated. The compiled analyses establishes [A/T][C/G]TTTTT[G/A]A[T/A] GTGT[T/C][T/C] as an MHV68 consensus for RTA recognition (Figure 6C).

## 3. Discussion

We and others have previously reported that NF-κB signaling impacts the lytic or latent outcomes of murine gammaherpesvirus 68 infection [26,27,30,35,37], but the mechanism is not well-defined. We developed tools to test the role of toll-like receptor activation of NF-κB signaling in promoting reactivation from latency in B cells. We generated a latent B cell line inducible for RTA-Flag expression. RTA expression alone drove a small degree of viral reactivation from latency that was significantly increased upon LPS treatment; but ORF6p activation was independent of the integrity of the NF-κB binding sites. RTA recognition elements previously predicted in the right oriLyt were identified by ChIP of the induced RTA-Flag. Generation of a recombinant RTA Bio-tagged virus permitted validation of RTA occupancy on the right oriLyt in the context of reactivation from primary B cells. LPS treatment of primary splenocytes from infected mice enhanced streptavidin-pull-down of RTA-bio complexes with the oriLytR region that spans three predicted RTA recognition elements.

### 3.1. Role of NF-κB Subunits

Disruption of NF-κB signaling upon Bay-11-7082 treatment of latent EBV+ or KSHV+ infected B cell lines induces reactivation [25]. It has been proposed that NF-κB signaling promotes latency via interference with RTA transactivation of lytic genes. For example, the NF-κB p65 subunit inhibits transactivation of lytic luciferase reporters by RTA proteins of MHV68, KSHV and EBV [25]. In addition, p65 antagonizes KSHV RTA activation of viral promoters that are co-regulated by RTA and RBP-Jκ via interference with protein-DNA complex formation [47]. Here, we report that co-transfection of NF-κB subunits p65, p50, or cRel with RTA had an inhibitory effect on RTA-mediated transactivation of the ORF6p reporter in 293T cells. However, NF-κB subunit inhibition of RTA transactivation occurred independently of the NF-κB recognition elements identified in the ORF6p, suggesting that NF-κB inhibition of RTA transactivation may occur instead by protein-protein interactions or by inducing the expression of other cellular repressors. RTA was not found in complex with the minimal RTA responsive region of the ORF6p that lacks an RRE, suggesting RTA induces the ORF6p via indirect action. Since *ORF6* is an early gene we attempted to perform chromatin immunoprecipitation for RTA at 6 h post-doxycycline treatment, but RTA protein was not consistently detectable in the HE-RIT cells at this timepoint (data not shown).

RTA binds p65 and promotes its ubiquitination and degradation [32], the p65 subunit has the potential to directly interact and interfere with RTA transactivation of viral genes. Perhaps a certain threshold of NF-κB p65 that inhibits RTA function in some contexts must be overcome to enable RTA to transactivate viral genes. As for the NF-κB subunits p50 and cRel that are found in the nucleus of MHV68 latently-infected cells, further experiments are required to test for interactions with RTA and to determine the functional consequences for RTA. A direct role for NF-κB subunits in regulating viral gene expression may be revealed only where NF-κB binding sites overlap with genomic regions occupied by RTA or co-factors [47].

Truncation of a portion of the ORF6 promoter that included the proximal NF-κB recognition site revealed an enhanced response to RTA in latent B cells that was not evident in 293T cells. This suggests that the inhibitory role of NF-κB signaling could be a context-dependent phenomenon. Grossman et al. [52] reported that the impact of NF-κB signaling in KSHV latency depends on the cellular context. Inhibition of NF-κB signaling impaired KSHV latency in BCBL-1 cells and promoted higher levels of lytic replication upon de novo infection of endothelial cells, yet was dispensable for the viral life cycle in human foreskin fibroblasts. KSHV lytic replication in endothelial, epithelial, and fibroblast cells was associated with higher levels of NF-κB activation [52,53] as we observed for MHV68 late during fibroblast infection [30]. Taken together, the impact of NF-κB subunits is likely cell type-specific due to the variation in active subunits and status of other transcription factors that is shaped by the microenvironment of the infected cell.

### 3.2. TLR4 Signaling Enhances Reactivation from Latency

TLR4 and TLR9 engagement by LPS or CpG DNA, respectively, increases MHV68 reactivation from latent B cells in cell culture and in mice [35,39]. We observed an enhancement in both viral DNA replication and lytic genes of early and late kinetic classes when LPS was combined with RTA induction in the HE-RIT cell lines. Curiously, agonists of TLR7 and TLR9 have been reported to repress MHV68 reactivation in cell culture [36], while agonists of TLR7 and TLR8 induce KSHV reactivation from latency [54]. A comprehensive analysis of a panel of TLR agonists in the HE-RIT system may provide insight into the host signaling pathways and impact on RTA function that led to differential outcomes of infection.

LPS induces multiple signaling pathways that may lead to synergistic transcription factor interactions with RTA or modifications of RTA to license MHV68 reactivation. With regard to upstream NF-κB signaling, IKKβ phosphorylation of RTA enhances RTA transactivation and a mutant virus lacking RTA phosphorylation sites is impaired for lytic replication [31,32]. Hence, the mechanism by which LPS activation of NF-κB signaling enhanced RTA transactivation of the ORF6 promoter may result from IKKβ phosphorylation of RTA. To address this, we treated HE2-RIT cell lines with doxycycline and LPS, or in combination with SC514, a selective inhibitor of IKKβ kinase activity [55]. We observed a loss of LPS-enhanced reactivation in cells treated with SC514 (data not shown), consistent with a role for IKKβ phosphorylation of RTA. However, our interpretation of this data was confounded by the significant loss of cell viability that accompanied the SC514 treatment (data not shown).

Genome-wide expression analysis identified two groups of viral genes that were directly responsive to RTA. However, most viral genes required both RTA and LPS for maximal expression. Since this full engagement of the lytic cascade comprising late genes remained dependent on viral DNA replication, LPS is likely enhancing IE or E gene expression. Thus, LPS may induce epigenetic changes that lead to activating histone modifications that potentiate RTA transactivation, possibly enhancing RTA occupancy of RRE in the viral genome. The oriLyt-R region examined here was one of numerous genomic regions found to associate with histone 3 in the context of lytic infection [56].

### 3.3. RTA Occupancy of Origins of Lytic Replication

Viral transcription factors influence the initiation of replication at the oriLyts of the gammaherpesvirus genomes. Similar to EBV and KSHV [57,58,59], MHV68 contains two functional oriLyt sequences that each serve as sites for initiating viral DNA replication [60,61]. The oriLyt-L and oriLyt-R have non-redundant roles for replication in specific cell types and tissues in vivo [61]. Binding of the EBV Zta protein to the oriLyt is essential for the initiation of DNA replication [62]. For KSHV, the K8 protein is functionally analogous to the EBV Zta protein as it interacts with the core replication machinery in the oriLyt to promote viral DNA replication [58,63]. KSHV RTA binds sites in its cognate oriLyts that are essential for viral DNA replication and recruits viral replication factors [64,65,66]. RTA binding and transcription in the KSHV oriLyt generates a polyadenylated RNA (Ori-RNA). During KSHV reactivation from RTA-inducible latent BCBL1 cells, RTA was preferentially recruited to the Ori-RNA promoter during reactivation [67]. Similarly, the MHV68 left origin of lytic replication contains two RTA responsive elements, RRE-A and RRE-B, which bind RTA [16]. The RRE-B site is necessary for transcription of the adjacent ORF18 gene [16]. Mutation of RRE-B disrupts expression of the essential ORF18 gene and impairs virus replication.

The RRE-A and RRE-B binding elements of the left oriLyt displayed homology to three other elements predicted in the right oriLyt, RRE-C, RRE-D, and RRE-E [16]; however, RTA occupancy had not been evaluated in the right oriLyt. Here, we confirm RTA occupancy of genomic regions spanning RRE-C and RRE-D and -E in the right oriLyt during MHV68 reactivation from latency, thus expanding the known targets of MHV68 RTA. Importantly, RTA occupancy of the right oriLyt region spanning RRE-D and RRE-E was independently observed in both latent B cell lines in cell culture and primary splenocytes isolated from the infected animal. LPS treatment led to enhanced occupancy at 18 h after explant. The close spacing between RRE-C, RRE-D and RRE-E in the right oriLyt raises the possibility that RTA occupancy of one site could contribute to the pull-down of the adjacent site. Further examination by EMSA with purified RTA or mutational analyses in the context of the viral genome will provide higher resolution of the importance of the individual RREs.

We generated a consensus motif based on the RRE elements of the MHV68 oriLyt regions and identified two additional RREs in the MHV68 genome, outside of the oriLyts. The RRE identified in the ORF9 promoter is occupied by RTA and mediates RTA transactivation [51], but the RRE in the ORF21 promoter has not been validated. Notably, this RRE is distinct from other RTA-responsive elements reported for MHV68 [18] and KSHV [12]. Interestingly, transcript levels of the MHV68 genes adjacent to the right oriLyt, *ORF*s *72*, *73*, *74*, and *M11*, were strongly induced by RTA alone. RTA of KSHV binds and activates the LANA promoter [68,69], but the LANA promoter of MHV68 initiates near or within the terminal repeats [70]. Mutagenesis of RRE in the right oriLyt is required to determine if RTA binding influences viral DNA replication and/or transactivation, perhaps via higher-order structures of the viral genome.

### 3.4. Conclusions

We designed and characterized two distinct systems to analyze RTA during reactivation from B cell latency, the HE-RIT B cell line inducible for reactivation by RTA and a recombinant virus that enables isolation of RTA from primary splenocytes of an infected mouse. RTA was found to occupy the right oriLyt in both of these latent B cell systems. LPS enhanced lytic gene expression of many viral genes such as ORF6, viral DNA replication and the frequency of reactivation in the HE-RIT B cell system. We find that the NF-κB sites in the ORF6 promoter do not impact RTA transactivation or the LPS enhancement of transactivation during productive infection. These two novel tools will enable whole genome analysis of RTA occupancy and the identification of RTA binding partners in the contexts of latency and reactivation from latency in cell culture, and during multiple aspects of gammaherpesvirus infection in primary latency reservoirs of the host.

## 4. Materials and Methods

### 4.1. Cells and Mice

Primary murine embryonic fibroblasts (MEFs), NIH3T12 murine fibroblasts and HEK293T cells were maintained in Dulbecco’s Modified Eagle Medium (DMEM) supplemented with 100 U/mL of penicillin and 100 mg/mL of streptomycin at 37 °C in 5% CO_2_. NIH3T12 were maintained in 8% fetal bovine serum (FBS) while MEFs and HEK293T cells were maintained in 10% FBS. S11 lymphoma [71], A20-HE2.1 [40], and A20-HE-RIT (described below) were maintained in RPMI 1640 supplemented with 10% FBS, 100 U/mL of penicillin, 100 mg/mL of streptomycin, and 50 mM 2-mercaptoethanol at 37 °C in 5% CO_2_. A20-HE2.1 cells were cultured in RPMI containing 300 µg/mL hygromycin B sulfate (Invivogen, San Diego, CA, USA). A20-HE-RIT cells were cultured in 300 μg/mL of hygromycin B sulfate, 300 μg/mL G418, and 2 μg/mL puromycin (Invivogen).

C57BL/6 mice were purchased from Jackson laboratories (Bar Harbor, Maine) or bred in the animal facilities at Stony Brook University. ROSA26HABirA mice ubiquitously express BirA, an HA-tagged bacterial biotin ligase (http://jaxmice.jax.org/strain/010920.html). The ROSA26HABirA mice (herein referred to as ROSA BirA mice) were a gift from Dr. Ming Li (Memorial Sloan Kettering Cancer Center, New York, NY, USA) and were bred in the Division of Laboratory and Animal Resources at Stony Brook University. All protocols were approved by the Institutional Animal Care and Use Committee of Stony Brook University.

### 4.2. Plasmids

The ORF6p luciferase reporter contains the putative regulatory region of the ORF6 gene (genomic coordinates 9894–11,218 bp, GenBank accession no. U97553) cloned into the HSP70-luc firefly luciferase reporter vector [41]. Mutation of NF-κB binding sites in the promoter of ORF6 (ORF6p) was performed using a Stratagene QuikChange II site-directed mutagenesis kit (Agilent Technologies, Santa Clara, CA, USA) according to the manufacturer’s protocol, and primers described in Table S1. For the -765 bp, -525 bp, and -331 bp, truncation mutants, PCR products were amplified from the ORF6p vector using the ORF6p reverse primer and forward primers described in Table S1. PCR products were digested and cloned into the SacI and XhoI sites of the HSP70-luc firefly reporter vector, courtesy of Dr. David Lukac (Rutgers Medical School, Newark, NJ, USA) [72]. NdeI and SacI digestion of the full length ORF6p in HSP70-Luc followed by *Klenow* blunting and ligation of the vector was performed to generate the -909 bp truncation mutant. To generate the -136 bp truncation mutant, the ORF6p vector was digested with HindIII, blunted by *Klenow* blunting, and digested with XhoI prior to ligation into the SmaI and XhoI sites of HSP70-luc. The -58 bp truncation mutant was generated by annealing the synthetic oligonucleotides described in Table S1.

p65, cRel, and p50 pcDNA3 expression vectors were gifts from Dr. Stephen Smale (Addgene plasmid# 20012, 20013, 20018) [73]. pCMV-IκBαM expression vector encodes the mutant superrepressor of IκBα with serine-to-alanine mutations at amino acids 32 and 36 (Stratagene, San Diego, CA, USA). The pCMV empty vector was generated by BamHI and HindIII digestion of pCMV-IκBαM to excise the IκBαM insert, *Klenow* blunting and religation. The RTA expression vector (pSG50) and empty vector (pCMV-Tag2B) were gifts from Dr. Samuel H. Speck (Emory University, Atlanta, GA, USA) [13].

### 4.3. Generation of Doxycycline Inducible B Cell Lines

A20-HE2.1 B cells are latently infected with a recombinant MHV68 that encodes a hygromycin resistance and enhanced green fluorescent fusion protein [40]. HE2.1 B cells were nucleofected with the pTRE3G-EF1α vector encoding the Tet-On 3G transactivator protein (Clontech, Mountain View, CA, USA). 24 h post-nucleofection cells were treated with 300 μg/mL G418 and hygromycin B sulfate (Invivogen, San Diego, CA, USA). Serial dilution of the cells into 96-well plates was performed six days later in the presence of G418 and hygromycin B sulfate to isolate individual cells. Clones from individual cells were screened for inducible expression of luciferase 24 h after nucleofection with the pTRE3G-luc vector and doxycycline treatment (5 μg/mL); the cell line clone (C10) had the highest doxycycline inducible activity.

Cloning of PGK-puro-pTRE3G-RTA-Flag-IRES-tdTomato began with PCR amplification of tdTomato from tdTomato vector (Invitrogen, Carlsbad, CA, USA) and cloning into EcoRV and BamHI sites of pTRE3G-IRES (Clontech). Next, FLAG-tagged RTA was amplified from pcDNA5-Topo-TA-RTA-Flag vector (a gift from Dr. Pinghui Feng, Keck School of Medicine of University of Southern California, Los Angeles, CA, USA) [31]. After PCR amplification of RTA-Flag, the product was digested with EagI, *Klenow* blunted, and digested with SalI. The product was ligated into pTRE3G-IRES-tdTomato that had been digested with BglII followed by *Klenow* blunting and SalI digestion. Last, PGK-puro was excised via EcoRV and EcoRI digestion from the pSico-PGK-puro vector, a gift from Dr. Tyler Jacks (Addgene plasmid# 11586) [74]. The excised PGK-puro construct was ligated into ZraI/Eco*R*I digested pTRE3G-RTA-FLAG-IRES-tdTomato vector, in the opposite orientation of transcription of the Tet3G transactivator initiated by EF1α.

Next, the HE2-C10 cells were nucleofected with PGK-puro-pTRE3G-RTA-FLAG-IRES-tdTomato construct and treated with 2 μg/mL puromycin (Invivogen), 300 μg/mL G418 and hygromycin B sulfate for eight days prior to serial dilution to isolate individual cells. After approximately three weeks colonies derived from single cells were validated for RTA induction by immunoblot. Two clones, designated RIT-G3 and RIT-F1, demonstrated robust RTA-Flag and tdTomato-Red expression in response to doxycycline treatment. For inductions, A20-HE2-RIT cells were subcultured 1:3 one day prior without drug selection. The following day the cells were seeded at 1.0 × 10^6^ cells/mL and treated with 5 μg/mL doxycycline and/or 20 ng/mL 12-O-tetradecanoylphorbol-13-acetate (TPA) (Sigma-Aldrich, St Louis, MO, USA). When examining enhancement by LPS, cells were treated with a range of doxycyline (2.5 ng/mL to 5 µg/mL) alone or in combination with 10 ng/mL to 20 µg/mL lipopolysaccharide (LPS) (Sigma-Aldrich), as indicated.

### 4.4. Generation of Recombinant Viruses

The MHV68 H2B-YFP genome was cloned into a bacterial artificial chromosome (BAC), kindly provided by Dr. Samuel Speck [75]. The biotinylation tag for RTA (Precission-FLAG-V5-TEV-TEV-Biotin acceptor) was a gift from Dr. Meinrad Busslinger (The Research Institute of Molecular Pathology, Vienna, Austria). The Precission-FLAG-V5-TEV-TEV-Biotin acceptor tag contains FLAG and V5 epitope tags that are flanked by a precission site and two sequential TEV sites, respectively. The biotin acceptor sequence follows the second TEV site. The biotinylation tag was amplified by PCR (primers described in Table S1) followed by digestion with XhoI and ApaI and ligation to XhoI and ApaI sites of pcDNA5-Topo-TA-RTA-FLAG to generate the RTA-Bio construct. The mCherry BirA ligase construct was a gift from Ralf Baumiester (Addgene plasmid# 23220) [76].

For generation of MHV68 H2BYFP-RTA-Bio, an intermediate was synthesized containing the RTA-Bio targeting construct with a kanamycin selection cassette and I-SceI site flanked by WT MHV68 sequence on either side (Genewiz, South Plainfield, NJ, USA). The product was excised from the pUC57 vector by EcoRV digestion and transformed into electrocompetent *E. coli* GS1783.5 harboring the MHV68 H2B-YFP BAC. After recovery, the cells were plated on chloramphenicol (30 μg/mL) and kanamycin (25 μg/mL) plates, at 30 °C for 48 h. The kanamycin selection marker was PCR amplified to verify insertion into the MHV68 H2B-YFP BAC. Using the protocol outlined previously [77], the kanamycin selection marker was removed via induction of the I-SceI homing enzyme, leaving behind the desired insertion. PCR amplification was performed to verify insertion of the Bio tag using primers described in Table S1. Two independent clones were isolated.

### 4.5. Analysis of Recombinant Viral BAC DNA

BAC DNA was prepared by column purification (Qiagen, Hilden, Germany). Restriction analysis was performed using 10 μg of BAC DNA digested overnight with MfeI or BamHI and then resolved in a 0.8% agarose gel in 0.5X Tris-acetate-EDTA. For complete genome sequencing, 1 μg BAC DNA was prepared for multiplex, 200-cycle, paired-end read sequencing using Nextera XT DNA Library Preparation Kit (Illumina, San Diego, CA, USA) with Miseq reagent kit v2 (Illumina) on an Illumina MiSeq by the Stony Brook Microarray Facility. Whole genome sequence data was aligned to the reference genome sequences using bowtie2 [78] in local alignment mode. Samtools mpileup with default options and vcfutils [79] were used to identify variants at depth coverage of over 1000.

### 4.6. Viruses and Plaque Assays

The recombinant MHV68 viruses were propagated as previously described [26,80]. For plaque assays performed on A20-HE2-RIT cell lines, inductions were performed with indicated drugs for 48 h and cell homogenates were generated by three freeze-thaw cycles. For growth curves, 1.0 × 10^5^ ROSA BirA MEFs were seeded in 12-well tissue culture plates prior to infection with recombinant MHV68 at a multiplicity of infection (MOI) of 5. Triplicate wells were harvested per time point, and cells with conditioned medium stored at −80 °C. NIH3T12 cells were seeded at 2 × 10^5^ cells per well in 6-well, and the next day were incubated with cell homogenate for 1 h prior to overlaying with 1.5% methylcellulose in DMEM containing 5% FBS. Nine days later, methylcellulose was removed and cells were washed twice with 1X PBS prior to methanol fixation and staining with 0.1% crystal violet solution in 10% methanol.

### 4.7. Antibodies and Immunoblot Analysis

For immunoblot analysis, cells were lysed in RIPA and whole cell lysates were resolved by SDS-PAGE and transferred to PVDF membrane. For RTA-Bio immunoblot analysis, HEK 293T cells were transfected with empty vector (pcDNA5-TOPO-TA) or RTA-Bio with or without the mCherry-BirA ligase expression vector using LT1 transfection reagent (Mirus Bio LLC, Madison, WI, USA) 72 h post-transfection cells were lysed in RIPA and immunoblot was performed. Immunoblot was also performed using ROSA BirA MEFs infected at MOI 5. Blots were probed with primary antibodies against FLAG (Sigma-Aldrich or Cell Signaling, Danvers, MA, USA), V5 (AbD Serotec, Raleigh, NC), GAPDH (Sigma-Aldrich) and ORF59 (Gallus Immunotech, Fergus, ON, Canada) [81]. Antibody against ORF65 (M9) was a gift from Dr. Ren Sun (University of California, Los Angeles, CA, USA) [82]. Secondary antibodies were goat anti-mouse (GE Healthcare), goat anti-rabbit (GE Healthcare), and goat anti-chicken (Gallus Immunotech) and anti-streptavidin (Rockland antibodies, Limerick, PA, USA), all conjugated to horseradish peroxidase. Chemiluminescent signals (ThermoScientific, Boston, MA, USA) were detected by audioradiography film or a LAS 500 Chemiluminesence Imager (GE Healthcare).

### 4.8. Quantitative PCR

Quantitative PCR was performed on A20-HE2-RIT cells after single or combination treatment with 5 μg/mL of doxycycline and/or 20 μg/mL of LPS, unless indicated otherwise. Total DNA from A20-HE2-RIT cells lines was column-purified (Qiagen) and input into a quantitative PCR reaction (SYBR green low ROX mix; Thermo Scientific) using primers for the viral ORF50 (forward, 5′-GGCCGCAGACATTTAATGAC-3′ and reverse, 5′-GCCTCAACTTCTCTGGATATGCC-3′); ORF46 (forward, 5′-GTCCCACCGAGCTTACACAA-3′ and reverse, 5′-GAACCAGGCCGTCCCTTATC-3′); or host GAPDH (forward, 5′-CCTGCACCACCAACTGCTTAG-3′ and reverse, 5′-GTGGATGCAGGGATGATGTTC-3′) genes. Copy number of viral genomes is calculated as fold increase in viral DNA copy number normalized to cellular GAPDH genomes over untreated cultures.

### 4.9. RNA Seq and Data Analysis

HE-RIT cells were treated with 5 µg/mL doxycyline (Dox) or a lower 0.5 µg/mL Dox in combination with 10 ng/mL LPS for 24 h. RNA was prepared by column purification (Qiagen RNeasy mini) and DNaseI digested. The sequencing library was prepared by BGI Americas (Cambridge, MA, USA). In brief, polyA mRNA was isolated from total RNA using poly (T) oligo-attached magnetic beads. The mRNA was fragmented and first-strand cDNA was generated using random hexamers, followed by second-strand cDNA synthesis and cDNA purification. The synthesized cDNA was subjected to end-repair and 3′ adenylated, followed by adaptor ligation, purification, and PCR amplification. The library was validating on the Agilent Technologies 2100 Bio-analyzer and the ABI StepOnePlus Real-Time PCR System prior to single-end 50 bp reads on an Illumina Hiseq4000 platform. The data discussed in this publication have been deposited in NCBI’s Gene Expression Omnibus and are accessible through GEO Series accession number GSE94238. (https://www.ncbi.nlm.nih.gov/geo/query/acc.cgi?acc=GSE94238).

For the analysis, RNA-seq reads from each dataset were first processed using Trimmomatic [83], where reads with low quality (<20) Phred score towards the leading or trailing ends were removed. Reads with lengths smaller than 20 were also removed. A joint reference was prepared from the annotated herpesvirus genome and the mouse transcriptome GRCm38 using RSEM [84]. Reads were then mapped to this reference using Salmon, version 0.7.2, with the k-mer size set to 19, to adjust to the shorter read length and the number of bootstraps for the variational Bayesian optimization set to 30 [85]. The average mapping rate for the six datasets was 89.76%. Quantification results from Salmon were imported into R using tximport [86], obtaining raw counts and TPM values summed to the gene level. The TPM values were scaled according to the length of the transcripts and the library size for each sample, using the length scaled option in tximport. The expression estimates were clustered based on the fold change of TPM across the samples. Genes that differed by more than two-fold between replicates were removed from the analysis. ORF50 was removed since expression was directly induced by doxycycline. Clustering was done in R using uncentered correlation as the similarity metric and complete linkage as the agglomeration method. Cluster (version 3.0) and Treeview (version 1.1.4r3) were used for the graphical output. Results are presented where the data is grouped into 5 clusters, with an average silhouette score of 0.61 [87].

### 4.10. Quantitative RT-PCR

Cells were lysed in RTL buffer (Qiagen) supplemented with 1% BME prior to total RNA column purification (Qiagen RNEasy Mini Kit) and DNase treatment (Ambion Turbo DNA Free kit) according to the manufacturers’ instructions. DNase-free RNA was reverse transcribed using SuperScript III (Invitrogen), and cDNA was analyzed by qPCR using PerfeCTa SYBR Green FastMix (Quanta Biosciences, Gaithersburg, MD, USA) in an ABI StepOne real-time PCR system (Applied Biosystems). PCR conditions were 95 °C for 15 min followed by 40 cycles of 95 °C for 15 s and 60 °C for 33 s. Primers for ORF26, ORF73, ORF46, ORF50, and GAPDH were described in [41] or above, and primers for ORF6 (forward, 5′-ATGTCTCCCCATATTCTTGC-3′ and reverse, 5′-ACATGGAAGTGTTGGCTGT-3′), ORF72 (forward, 5′-ACAACACTGGTGTAGCTCTTC-3′ and reverse, 5′-GGCCTGTATGCACTTAACTATG-3′), and ORF75C (forward, 5′-ATTCCAGAGTATTCGTTCAG-3′ and reverse, 5′-GTCAGCACTGTCCAATTCT-3′) were purchased from Eurofins genomics (Huntsville, AL, USA). The cycle threshold (CT) determination was within the linear range of detection for each amplimer. Relative quantitation was calculated based on the ΔΔCT method.

### 4.11. Electrophoretic Mobility Shift Assays

Transcription Element Search Software (http://www.cbil.upenn.edu/tess/) was used to search for candidate NF-κB binding sites in the MHV68 genome based on the consensus binding site (GGGAMTTYCC) allowing for a one nucleotide mismatch in the consensus sequence. For nuclear extracts, MHV68+ latent S11 B lymphoma cells or lytic infected MEF cells (MOI 5) were harvested and washed once in cold PBS. The cell pellet was resuspended in ice cold hypotonic lysis buffer (10.0 mM HEPES pH 7.9, 10.0 mM KCl, 1.5 mM MgCl_2_, 0.1 mM EDTA, 1.0 mM DTT, 0.5 mM PMSF, one mini EDTA-free proteinase inhibitor cocktail (Roche, Basel, Switzerland), and left on ice 15 min. Next, NP40 was added and cells were vortexed briefly before spinning at 10,000× *g* for 5 min at 4 °C. The nuclei pellet was washed with hypotonic buffer and resuspended in high salt buffer (25% glycerol, 20 mM HEPES pH 7.9, 420 mM NaCL, 1.5 mM MgCl_2_, 0.2 mM EDTA, 0.5 mM DTT, 0.5 mM PMSF, EDTA-free proteinase inhibitor cocktail) with vigorous shaking for 2–3 h at 4 °C before centrifugation at 10,000× *g* for 10 min at 4 °C.

For analysis of direct binding, a ^32^P-labeled oligonucleotide containing the underlined NF-κB consensus site, 5′-AGTTGAGGGGACTTTCCCAGGC-3′, was incubated at room temperature for 30 min with 2.5 μg of nuclear extracts in binding buffer (2.0 mM HEPES pH 7.9, 1.0 mM EDTA, 5.0 mM DTT, 0.05% Triton X-100, 5% glycerol, and 2.0 μg poly dI-dC). For cold competition analysis, unlabeled oligonucleotides containing putative WT or mutant NF-κB binding sites were added at 10- and 100-fold molar excess of labeled probe. Nucleoprotein complexes were run in 5% native polyacrylamide gels at 190 V, dried under vacuum, and imaged using the Storm 840 PhosphoImager (GE Healthcare, Piscataway, NJ, USA). For EMSA supershifts, overnight incubation of nuclear extracts with 0.2 to 2.0 μg of antibodies against NF-κB subunits (all purchased from Santa Cruz, Dallas, Texas): p65 (sc-109x), (sc-114x), cRel (sc-71x), RelB (C-19x), p52 (K-27x), IgG rabbit (sc-2027) was performed at 4 °C prior to incubation with nuclear extracts.

### 4.12. Luciferase Reporter Assays

To examine the minimal regulatory region of the ORF6 promoter, HEK 293T cells were seeded into 24-well plates one day prior to calcium phosphate transfection with 1.0 μg of the full length ORF6p luciferase reporter or promoter mutants along with either 0.5 μg RTA (pSG50) or empty vector (EV, pCMVTag2B) and 355 ng pRL-TK [13,41]. At 48 h post-transfection the cells were lysed in 1X passive lysis buffer (Promega, Madison, WI) and luciferase assay performed using pRL-TK to normalize. For NF-κB subunit co-transfection experiments in HEK 293T cells, plasmids expressing NF-κB subunits p50, cRel or p65 or pcDNA3 empty vectors were transfected at 1:1 ratios with pSG50. Protein content was used for normalization.

For ORF6p analysis in HE2 B cells, 2.0 × 10^6^ cells were nucleofected, using electroporation solution (Mirus Bio LLC, Madison, WI), with 1.0 μg of either the full length ORF6p luciferase reporter or ORF6p truncation mutants and 0.5 μg RTA-Flag (RTA-FLAG-pcDNA5-Topo-TA) or EV (pCDNA5-Topo-TA) for 24 h. For NF-κB superrepressor experiments, HE2 B cells were nucleofected with 0.5 μg full length ORF6p reporter vector or the double NF-κB site mutant (ORF6p DM), 0.25 μg RTA-Flag (RTA-FLAG-pcDNA5-Topo-TA) or EV (pCDNA5-Topo-TA) and 0.25 μg IκBαM (pCMV-IκBαM) or pCMV empty vectors. To verify NF-κB signaling components, nucleofection was performed using pGL-4.32, 0.25 μg of p65 or 0.25 μg IκBαM (pCMV-IκBαM), and luciferase values were normalized to protein content.

To examine ORF6p transactivation in the context of infection, NIH3T12 cells were seeded at 8.0 × 10^5^ cells per 10 cm dish one day prior to transfection with 15 μg pGL4.32 or ORF6p-luc by Superfect (Qiagen). The next day 1.0 × 10^5^ cells per well were seeded into 12-well plates and then infected the following day with MHV68-IκBαM or MHV68-IκBαM.MR [26] at a multiplicity of infection (MOI) of 5. Luciferase assays were performed 24 hpi.

### 4.13. Chromatin Immunoprecipitation

Chromatin immunoprecipitation was performed using EZ-Magna ChIP G kit (EMD Millipore, Billerica, MA, USA). Briefly, 1.0 × 10^7^ A20-HE2-RIT cells were induced with 5 µg/mL doxycycline alone, or in combination with 20 µg/mL LPS. Primary splenocytes were resuspended at 6.0 × 10^6^ cells/mL in 10 mL DMEM and treated with 100 ng/mL LPS for 18 h. After treatment, cross-linking was performed with formaldehyde, terminated with glycine, and the cells were washed twice with 1X PBS containing protease inhibitors. Cell lysis was performed and nuclear extracts were resuspended in 0.5 mL of nuclear lysis buffer. Nuclear lysates were sonicated to ~100 bp to 500 bp fragments using a Branson Sonifier 450 (Danbury, CT, USA). Sonicates were centrifuged at 10,000× *g* at 4 °C for 10 min to remove insoluble material. Lysates were pre-cleared with protein A/G agarose (Life Technologies, Grand Island, NY, USA) slurry for 30 min at 4 °C. Immunoprecipitation was performed overnight with anti-FLAG M2 magnetic beads (Sigma-Aldrich) or streptavidin conjugated beads. Immunocomplexes were washed sequentially using EZ-Magna ChIP low salt immune complex buffer, high salt immune complex buffer, LiCl immune complex buffer, and TE buffer. Elution was performed using the provided EZ-Magna ChIP elution buffer, followed by proteinase K digestion overnight at 62 °C. DNA was recovered by phenol-chloroform extraction and ethanol precipitation.

DNA samples were analyzed for viral genomic regions of interest using primers described in Table S1 in a 384-well qPCR Roche LightCycler 480 machine (Roche, Basel, Switzerland). Technical replicates of three to five biological replicates were analyzed for each condition and normalized by 1% input DNA. Immunoprecipitates recovered were analyzed by qPCR to amplify ORF6p, ORF65 RREC and RRED-E (sequences described in Table S1).

### 4.14. Infections and Organ Harvests

Eight- to ten-week-old ROSA26HABirA mice were infected by intraperitoneal injection with 1000 PFU of either MHV68 H2B-YFP or MHV68 H2B-YFP-RTA-Bio viruses under isoflurane anesthesia. Inoculum titers were determined to confirm infectious dose. Mice were sacrificed by the application of terminal isoflurane anesthesia. For latency, reactivation, and ChIP experiments, mouse spleens were homogenized and treated with Tris-buffered ammonium chloride to remove red blood cells, and then filtered through 100 μm pore-sized nylon. For peritoneal cells, 10 mL of media was injected into the peritoneal cavity, agitated, and withdrawn using an 18-gauge needle. Peritoneal exudate cells were pelleted by centrifugation and resuspended in 1 mL of DMEM supplemented with 10% FBS.

### 4.15. Limiting Dilution PCR Detection of MHV68 Genome Positive Cells

For determining the frequency of cells harboring the viral genome, single cell suspensions of cells were prepared from mice and used in single-copy sensitive nested PCR. Six 3-fold serial dilutions of cells were plated into 96-well PCR plates in a background of 3T12 cells and lysed overnight with proteinase K at 56 °C. The plate was subject to an 80-cycle nested PCR with primers specific for MHV68 ORF50 [80]. At each serial dilution, twelve replicates were analyzed and plasmid DNA at 0.1, 1, and 10 copies included to verify single-copy sensitivity of the assay.

### 4.16. Limiting Dilution Ex Vivo Reactivation Assay

In order to determine the frequency of cells that harbor latent virus capable of reactivation, single-cell suspensions were prepared from mice 16 days post-infection, resuspended in DMEM supplemented with 10% FBS and plated in 12 serial 2-fold dilutions onto a monolayer of C57BL/6 MEFs in 96-well tissue culture plates [80,88]. Twenty-four replicates were plated per serial dilution and wells were scored for cytopathic effect 2–3 weeks after plating. Parallel samples were mechanically disrupted using a mini-bead beater with 0.5 mm beads prior to plating on a monolayer of MEFs to release preformed virus to differentiate between preformed infectious virus and virus spontaneously reactivating upon explant.

### 4.17. Motif Analysis

A fixed-size 22-bp Position Weight Matrix (PWM) was generated from known RRE-A and RRE-B RTA binding sites [16] using Glam2 [89]. RTA consensus recognition elements in the MHV68 genomes were located with Find Individual Motif Occurrences (FIMO) utility with the *p*-value cutoff set at 1 × 10^−6^ [90]. The background DNA zeroth-order Markov probabilities were generated by FIMO from a non-redundant database [91].

### 4.18. Statistical Analyses

Data was analyzed using Prism 5 software (GraphPad, La Jolla, CA, USA). Statistical significance was determined using Student’s *t*-test or one-way ANOVA followed by Tukey’s multiple comparison test. Under Poisson distribution analysis, the frequency of latency establishment and reactivation from latency was the intersection of nonlinear regression curves with the line at 63.2, the significance was determined by paired *t*-test.

## Supplementary Materials

The following are available online at www.mdpi.com/2076-0817/6/1/9/s1, Figure S1: Characterization of a MHV68+ latent B cell line inducible for reactivation; Figure S2: NF-κB subunits that bind to the ORF6 regulatory region vary with the type of infected cell nuclear extract; Figure S3: RTA activation of the ORF6 promoter is independent of NF-κB subunit interaction with the recognition sites; Figure S4: RTA-Bio virus replication is impaired in mouse embryonic fibroblasts; Figure S5: The RTA-Bio virus colonizes B cells similar to the control virus, but has a latency defect in the peritoneal exudate cells; Table S1: Oligonucleotides used for cloning and ChIP-PCR.
