# Supplementary material for: RTA Occupancy of the Origin of Lytic Replication during Murine Gammaherpesvirus 68 Reactivation from B Cell Latency
Source: Pathogens. 2017 Feb 16;6(1):9. doi: 10.3390/pathogens6010009 (PMC5371897; doi:10.3390/pathogens6010009)
Supplement: Supplementary file 1 [file pathogens-06-00009-s001.pdf]

pathogens

Supplementary of RTA Occupancy of the Origin of Lytic Replication during Murine gammaherpesvirus 68 Reactivation from B Cell Latency

Alexis L. Santana [1,2], Darby G. Oldenburg [3], Varvara Kirillov [2], Laraib Malik [4], Qiwen Dong [5], Roman Sinayev [6], Kenneth B. Marcu [2,7,8,9], Douglas W. White [3] and Laurie T. Krug [2,*]

1 The Ronald O. Perelman Department of Dermatology, New York University School of Medicine, New York, NY 10016, USA; Alexis.santana@nyumc.org (A.L.S.)
2 Department of Molecular Genetics and Microbiology, Stony Brook University, Stony Brook, NY 11794, USA; varvara.kirillov@stonybrook.edu (V.K.); kenneth.marcu@stonybrook.edu (K.B.M.)
3 Gundersen Health System, La Crosse, WI 54601, USA; darby.oldenburg@gmail.com (D.G.O.); DWWhite@gundersenhealth.org (D.W.W.)
4 Department of Computer Science, Stony Brook University, Stony Brook, NY 11794, USA; laraib.malik@stonybrook.edu
5 Program in Molecular and Cellular Biology, Stony Brook University, Stony Brook, NY 11794, USA; qiwen.dong@stonybrook.edu
6 Applied Mathematics and Statistics, Stony Brook University, Stony Brook, NY 11794, USA; roman@rsinayev.com
7 Biomedical Research Foundation Academy of Athens (BRFAA), Athens 115 27, Greece
8 Biochemistry and Cell Biology Dept., Stony Brook University, Stony Brook, NY 11794, USA
9 Dept. of Pathology, Health Sciences Center, Stony Brook University, Stony Brook, NY 11794, USA
* Correspondence: laurie.krug@stonybrook.edu; Tel.: +1-631-632-9055

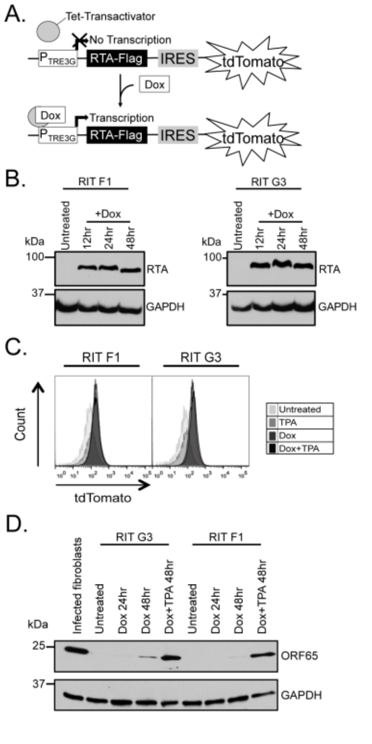

Figure S1. Characterization of a MHV68+ latent B cell line inducible for reactivation. (A) Schematic of reactivation inducible HE2 latent B cells lines (HE-RIT) whereby doxycycline (Dox) treatment induces expression of Flag-tagged RTA and tdTomato fluorescent protein. (B) Western blot of Flag-tagged RTA expression from two individual HE-RIT clones (F1 and G3) at indicated hours post-dox treatment. (C) Flow cytometry analysis of tdTomato fluorescence upon Dox or TPA treatment, alone or in combination for 24 h. (D) Validation of a lytic antigen expression in MHV68+ latent B cell line inducible for reactivation. Immunoblot detection of lytic antigen expression after doxycycline induction of HE-RIT cell lines at the indicated times.

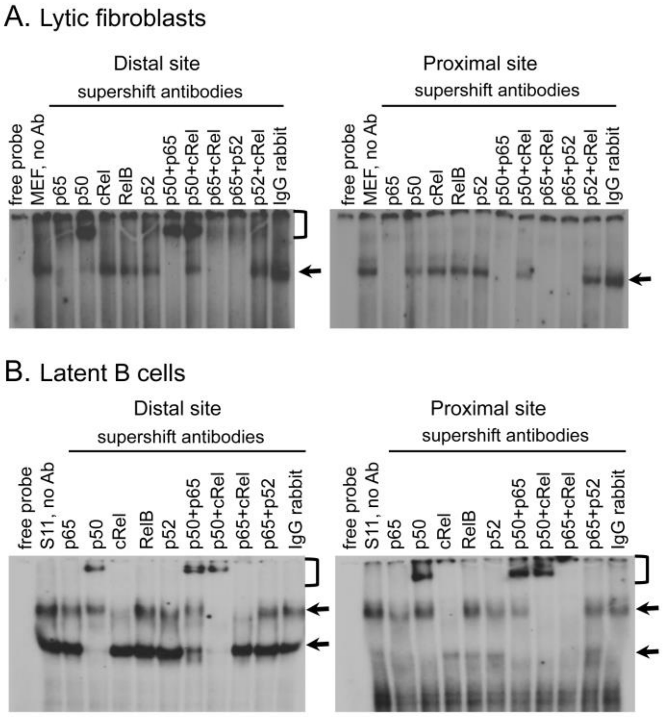

Figure S2. NF-κB subunits that bind to the ORF6 regulatory region vary with the type of infected cell nuclear extract. (A) EMSA supershift analysis was performed using ^{32}P-labeled ORF6p distal and proximal NF-κB site oligonucleotides, nuclear extracts from MHV68+ infected mouse embryonic fibroblasts and the indicated antibodies. (B) EMSA supershift analysis was performed using 32P-labeled ORF6p distal and proximal NF-κB site oligonucleotides, nuclear extracts from latent MHV68+ S11 B cells and the indicated antibodies. Arrows indicate shifts and brackets denote supershifts.

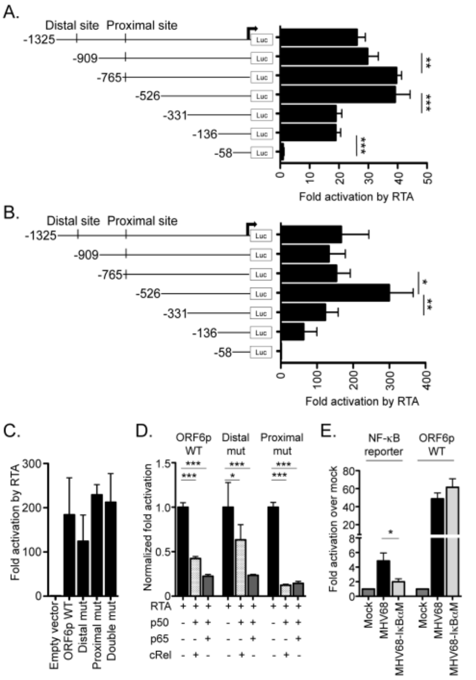

Figure S3. RTA activation of the ORF6 promoter is independent of NF-κB subunit interaction with the recognition sites. (A) Schematic diagram of 5′ deletions of the ORF6p firefly luciferase reporter. Transcriptional activation by RTA was analyzed in latent MHV68+ HE2 B cells. Data is normalized by protein content and shown as fold activation by RTA over reporter alone. (B) Schematic diagram of 5′ deletions of the ORF6 promoter (ORF6p) used for reporter assays. Transcriptional activation by RTA was analyzed in HEK293T cells at 48 h. ORF6p truncation mutants were transfected with the RTA expression vector or empty vector. Data is normalized by renilla luciferase and shown as fold activation by RTA over reporter alone +/- SD; *, p<0.05; **, p<0.01, one-way ANOVA, post-hoc Tukey's test. (C) Transcriptional activation by RTA of the full-length WT ORF6p or NF-κB site mutations in HEK293T cells. ORF6p NF-κB site mutants were transfected with the RTA expression vector or empty vector. Data is normalized by renilla luciferase and shown as fold activation by RTA over empty luciferase reporter +/- SD. (D) Co-transfection of HEK293T cells with either the ORF6p or NF-κB site mutant reporter constructs RTA and NF-κB subunits luciferase values were normalized to protein content and shown relative to RTA transactivation alone +/- SD; *, p<0.05; ***, p<0.001, one-way ANOVA, post-hoc Tukey's test. (E) NIH3T12 cells were transfected with NF-κB or ORF6p reporters followed by infection with control MHV68 (MOI 5.0) WT or MHV68-IκBαM, that expresses the NF-κB super-repressor IκBαM. Luciferase assays were performed 24 h post-infection. Bars represent fold activation relative to mock infected cells for triplicate samples +/- SD; *, p=0.013, unpaired t-test.

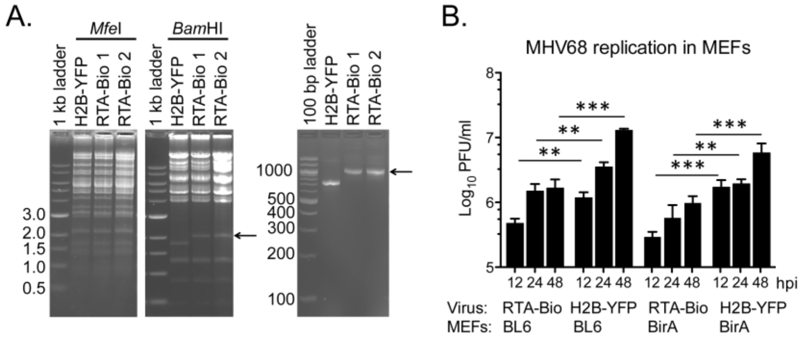

Figure S4. RTA-Bio virus replication is impaired in mouse embryonic fibroblasts. (A) Restriction fragment length polymorphism analysis of two independent clones of RTA-Bio upon *Mfe*I or *Bam*HI digestion of BAC DNA (left panels). Arrow denotes expected change in banding pattern upon Bio-tag insertion. PCR amplimer size shift confirms insertion of Bio-tag (indicated by arrow) in the RTA-Bio BAC DNA (right panel). (B) Virus growth in primary MEFs isolated from C57/BL6 (BL6) or Rosa BirA (BirA) mice upon infection with H2B-YFP or RTA Bio viruses (MOI 5.0). Bars indicate mean values +/- SD; **, p<0.01; ***, p<0.001, one way ANOVA, post-hoc Tukey's test.

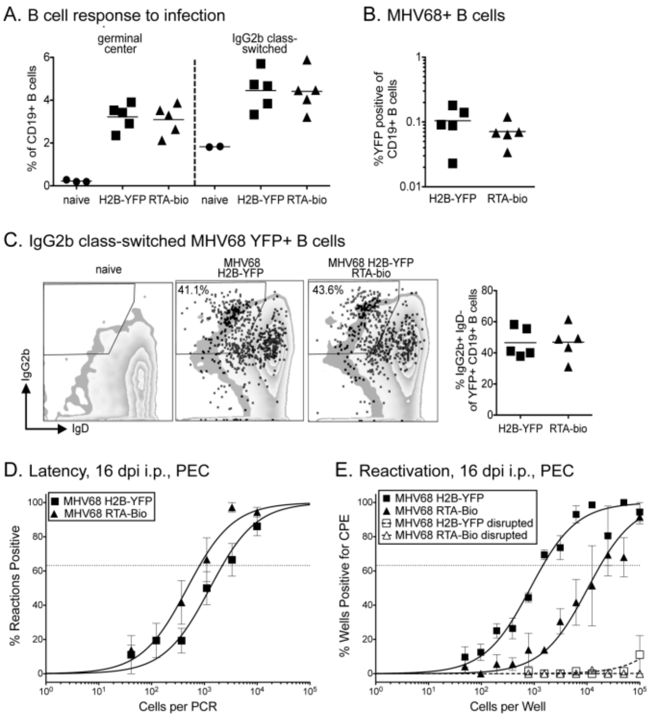

Figure S5. The RTA-Bio virus colonizes B cells similar to the control virus, but has a latency defect in the peritoneal exudate cells. ROSA BirA mice were infected with 1000 PFU of the designated viruses by the intraperitoneal route. (A) Percentage of CD19+ B cells positive for germinal center or immunoglobulin class switched to IgG2b. (B) Percentage of total CD19+ B cells positive for infection by the indicated viruses, measured by YFP expression. (C) Percentage of IgG2b class-switched B cells in the MHV68 YFP+ population of mice infected with the indicated viruses. Left panels, black dots indicate YFP+ cells and the gate indicates the percentage of YFP+ cells that are IgG2b+ IgD- B cells. The individual cells are overlaid on the flow cytometry plot of total CD19+ B cells. Representative flow cytometry plot of B cell response in naïve mice provided. Right panel, summary plot of the percentage of MHV68 YFP+ cells that bear the immunoglobulin class-switch markers IgG2b and IgD. (D) Latency was measured at 16 dpi in the spleen, as indicated by the frequency of intact splenocytes harboring the viral genome using a limiting-dilution nested-PCR assay. (E) Reactivation from latency was measured by using a limiting-dilution explant reactivation co-culture assay. Dotted lines represent disrupted cells used to measure preformed infectious virus. The intersection of the nonlinear regression curves with dashed line at 63.2% is used to determine the frequency of cells that were positive for either the viral genome or reactivating virus. Graphs represent SEM of three-five independent experiments using three-four mice each.

Table S1. Oligonucleotides used for cloning and ChIP-PCR

Gene target[a]	Genomic strand[b]	Primer Sequence[c]
**ORF6p distal site mutant**		5'-TGGAGTGGCCAATCTCCATATGT-3
**ORF6p distal site mutant**	R	5'-CATATGGGAGATTGGCCACTCCA-3'
**ORF6p proximal site mutant**	F	5'-TTTTGCCTCGAAATTCCCTGTGG-3'
**ORF6p proximal site mutant**	R	5'-CCACAGGGAATTTCGAGGCAAAA-3'
**ORF6p -765bp truncation mutant**	F	5'-GATCGAGCTCGTGAGGGACCCGGGTGGACA-3'
**ORF6p -526bp truncation mutant**	F	5'-GATCGAGCTCGATGGGCACTCCTATGTGAC-3'
**ORF6p -331bp truncation mutant**	F	5'-GATCGAGCTCACGGCGTCCCCAGTCACTCA-3'
**ORF6p reverse truncation mutant**	R	5'-GATCCTCGAGCATGATGAGTGTCCAAAAGCAGAGAGG-3'
**ORF6p -58bp truncation mutant A**	F	5'-CAAAATCCCAATTCTCCTCA-3'
**ORF6p -58bp truncation mutant B**	F	5'-TGCTAGTCGCGTCCTCTCTG-3'
**ORF6p -58bp truncation mutant C**	F	5'-CTTTTGGACACTCATCATGC-3'
**ORF6p -58bp truncation mutant D**	R	5'-ATTGGGATTTTGAGCT-3'
**ORF6p -58bp truncation mutant E**	R	5'-CGACTAGCATGAGGAGA-3'
**ORF6p -58bp truncation mutant**	R	5'-CCAAAAGCAGAGAGGACG-3'

**F**		
**ORF6p -58bp truncation mutant G**	R	5'-TCGAGCATGATGAGTGT-3'
**tdTomato**	F	5'-GATCGATATCATGGTGAGCAAGGGCGAGGAG-3'
**tdTomato**	R	5'- GATCGGATCCGGCACAGTCGAGGCTG-3'
**Flag-tagged RTA**	F	5'-GATCGTCGACATGGCCTCTGACTCGGATTCC-'3
**Flag-tagged RTA**	R	5'-GATCCGGCCGCAGTAGCAGCAGGA-3'
**Bio-tag**[d]	F	5'-<br>GAATTCGGCCGGCCATGCATTTACAATTGGGCGGTGG<br>AGGTCTGGAAGTTCTGTTCC<br>AGGGACCTGACTACAAGGACGATGACGATAAAGGGAA<br>GCCAATCCCTAATCCCCTTCTGG<br>GACTCGACTCTACCGAAAACTTGTACTTCCAGGGACCA<br>CGGGAAAATCTGTACTTTCAGG<br>GAATGGCATCGAGTCTACGGCAAATCCTCGACTCGCA<br>GAAGATGGAGTGGCGCTCAAACG<br>CCGGAGGCTCGTGACCATGGCGCGCCGAATTC-3'.
**RTA-Bio**	F	5'-GATCCTCGAGGTTAACGGAATTCGGCC-3'
**RTA-Bio**	R	5'-GATCGGGCCCGCCATGGTCACGAGCCT-3'
**RTA-Bio BAC PCR**	F	5'-CCATTTTCACCCATTAGCCCT–3'
**RTA-Bio BAC PCR**	R	5'-ACTTAAGGATTTAGAAATGTCTTGT-3'
**ChIP-ORF6 (0-150)**	F	5'-AGACTCTGAAGTGCTGACTCGGC-3'
**ChIP-ORF6 (0-150)**	R	5'-GATGAGTGTCCAAAAGCAGAGAGGA-3'
**ChIP-ORF65**	F	5'-CGTCAGACATAGACCCTGGAT-3'
**ChIP-ORF65**	R	5'-TGGCCCTCTACCTTCTGTTGA-3'
**ChIP-RREC**	F	5'-GCCTGGGGAGCCAAAGCGAG-3'
**ChIP-RREC**	R	5'-GCAATAGGCCAGGTGGGCCG-3'
**ChIP-RRED-E**	F	5'-CGGACCAATCACCAACTTGACG-3'
**ChIP-RRED-E**	R	5'-TCGGTTTGCGGTTAGACCAGGC-3'

[a] Genomic target of PCR primer for cloning or qPCR

[b] F=Forward primer, R=Reverse primer

[c] Primer sequence used for PCR amplification

[d] Sequence of Bio tag inserted at the C-terminus of RTA, Flag (underlined), V5 (bold), Biotin acceptor sequence (bold and underlined)

© 2017 by the authors. Submitted for possible open access publication under the terms and conditions of the Creative Commons Attribution (CC BY) license (http://creativecommons.org/licenses/by/4.0/).
